# Automated Prediction of Crack Propagation Using H2O AutoML

**DOI:** 10.3390/s23208419

**Published:** 2023-10-12

**Authors:** Intisar Omar, Muhammad Khan, Andrew Starr, Khaled Abou Rok Ba

**Affiliations:** School of Aerospace, Transport and Manufacturing, Cranfield University, Bedford MK43 0AL, UK

**Keywords:** Acrylonitrile Butadiene Styrene (ABS), automated machine learning, H2O, crack propagation prediction, hyperparameter tunning

## Abstract

Crack propagation is a critical phenomenon in materials science and engineering, significantly impacting structural integrity, reliability, and safety across various applications. The accurate prediction of crack propagation behavior is paramount for ensuring the performance and durability of engineering components, as extensively explored in prior research. Nevertheless, there is a pressing demand for automated models capable of efficiently and precisely forecasting crack propagation. In this study, we address this need by developing a machine learning-based automated model using the powerful H2O library. This model aims to accurately predict crack propagation behavior in various materials by analyzing intricate crack patterns and delivering reliable predictions. To achieve this, we employed a comprehensive dataset derived from measured instances of crack propagation in Acrylonitrile Butadiene Styrene (ABS) specimens. Rigorous evaluation metrics, including Mean Absolute Error (MAE), Root Mean Square Error (RMSE), and R-squared (R^2^) values, were applied to assess the model’s predictive accuracy. Cross-validation techniques were utilized to ensure its robustness and generalizability across diverse datasets. Our results underscore the automated model’s remarkable accuracy and reliability in predicting crack propagation. This study not only highlights the immense potential of the H2O library as a valuable tool for structural health monitoring but also advocates for the broader adoption of Automated Machine Learning (AutoML) solutions in engineering applications. In addition to presenting these findings, we define H2O as a powerful machine learning library and AutoML as Automated Machine Learning to ensure clarity and understanding for readers unfamiliar with these terms. This research not only demonstrates the significance of AutoML in future-proofing our approach to structural integrity and safety but also emphasizes the need for comprehensive reporting and understanding in scientific discourse.

## 1. Introduction

Crack propagation is a critical phenomenon with far-reaching implications for structural integrity, reliability, and safety across a myriad of applications in materials science and engineering. Accurately predicting crack propagation behavior is pivotal to ensuring structural performance and the longevity of engineering components. However, traditional methodologies for crack propagation analysis, such as empirical observations, physical testing, and numerical simulations, are often labor-intensive, expensive, and require expert knowledge, which are significant deterrents to their broad applicability [1,2,3,4,5,6].

With the increasing demand for efficient and precise automated models to predict crack propagation, this paper presents an innovative approach that utilizes the H2O library to forecast crack propagation in ABS materials. ABS is a widely used thermoplastic known for its remarkable mechanical properties, including toughness, impact resistance, and thermal stability. Understanding crack propagation in ABS holds significant interest in industries such as automotive, aerospace, and consumer goods manufacturing. The objective of this research is to design a machine learning-based automated model capable of accurately predicting crack propagation behavior in ABS materials. The versatile H2O library, a Python tool for machine learning, offers a comprehensive range of algorithms, automated hyperparameter tuning, and feature engineering capabilities [7,8,9,10]. By leveraging these capabilities, the model can efficiently analyze complex crack propagation patterns and provide reliable predictions, thereby streamlining the process of model development and handling large datasets. This research contributes to the field of crack propagation prediction by demonstrating the effectiveness of an automated approach using the H2O library. Its aim is to enhance the efficiency and accuracy of crack propagation analysis, leading to improved assessments of structural integrity and maintenance strategies. Despite extensive research on crack propagation prediction methods, traditional approaches such as analytical methods and numerical simulations often struggle to capture the intricate dynamics of crack propagation [1,11,12,13,14,15].

Furthermore, machine learning techniques for damage and crack detection have shown promising results but require significant human intervention for feature selection, model selection, and hyperparameter tuning, limiting their widespread adoption [16]. In contrast, employing a deep neural network to forecast crack propagation in various materials holds tremendous potential for advancing the domain of materials science [17,18,19,20,21]. These advanced models possess the ability to scrutinize intricate patterns within extensive datasets, thereby assisting researchers in comprehending crack behavior across a wide range of materials. However, it is crucial to acknowledge a notable drawback inherent to deep neural networks: their computational intensity and the requirement for substantial amounts of data to achieve accurate predictions. This constraint often poses challenges, particularly when confronted with limited or moderately sized datasets. Hence, the astute selection of platforms like H2O assumes paramount importance, as they excel at efficiently handling datasets of varying sizes, be they small, moderate, or large. Consequently, researchers in material science and other disciplines grappling with diverse dataset sizes gravitate towards these platforms as their preferred choice. Considering these limitations, the emergence of AutoML platforms, such as the H2O library, provides a compelling solution. AutoML automates the end-to-end process of applying machine learning, including data pre-processing, feature selection, model selection, and hyperparameter tuning, minimizing the need for human intervention and expert knowledge.

While AutoML has found applications across diverse sectors, such as healthcare, finance, and retail [10,22,23,24,25,26,27,28,29,30,31,32], its application in structural health monitoring, particularly for crack and damage detection, remains underexplored. Moreover, although the H2O library possesses powerful capabilities, its usage in this specific use case has not been thoroughly investigated. Therefore, conducting a comprehensive study on the application of the H2O AutoML library for crack detection in engineering materials is crucial. This study not only contributes to the existing literature on the integration of AutoML in structural health monitoring but also provides valuable insights into the practical implications of employing the H2O library in this critical field of study. The promising potential of AutoML to revolutionize the process of crack and damage detection highlights the urgency of further research in this area. This study contributes to the field of crack propagation prediction by demonstrating the effectiveness of an automated approach using the H2O library. By automating the crack propagation prediction process, our goal is to enhance the efficiency and accuracy of crack propagation analysis, facilitating improved assessment of structural integrity and maintenance strategies. The following sections will discuss the methodology, experimental results, and implications of our research, followed by a comprehensive analysis of the findings. Through this work, we aim to advance the understanding of crack propagation in ABS materials and provide valuable insights for practical applications in engineering and materials science.

## 2. Methodology

### 2.1. Dataset Description and Preprocessing

**Figure 1:**
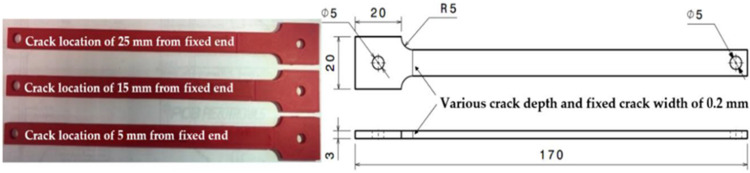
Specimen’s geometry dimensions in mm [33,34,35].

**Figure 2:**
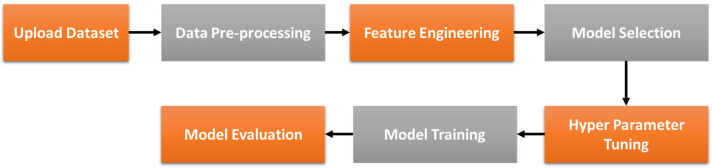
Flowchart of the process of the AutoML H2O Application.

The dataset is representative of typical crack propagation scenarios encountered in ABS materials. A variety of samples were manufactured using the geometry presented in Figure 1. In the process of conducting the experiments, the sample was secured to the vibrating platform and subjected to varying degrees of heat. This platform was then tasked with exerting mechanical forces, while impact tests were initiated to identify the primary vibrational frequency of the sample. A laser vibrometer was employed to capture measurements. Subsequently, a vibration test was implemented at this identified frequency. Should there be any manifestation of crack development, the amplitude of the beam tip’s displacement would decline, prompting a halt to the shaker in order to register the new frequency. Additional impact tests were carried out to ascertain this new frequency, which was subsequently programmed into the shaker. The cycle of these steps continued until the sample endured a catastrophic failure as a result of crack progression. The collected data includes information on various parameters (as shown in Table 1), such as temperature °C, crack location mm, amplitude mm, natural frequency Hz, and a predicted value: crack depth mm. The crack propagation measurements were obtained from previous studies [33,34,35]. Prior to model development (as shown in Figure 2), the data are processed and refined to ensure it is optimized for training the automated machine learning model.

Data preprocessing involves outliers, handling missing values, and data normalization. Outliers are identified and treated using robust statistical methods. Missing values are imputed using appropriate techniques, such as mean imputation or regression-based imputation.

Data normalization is performed to scale the features to a consistent range, reducing the impact of varying magnitudes. The dataset was randomly and blindly divided into training and test sets, employing a 70/30 split. Selecting a 70% training data portion was crucial to providing a substantial dataset for training the models selected via the H2O library. This extensive dataset facilitated the accurate capture of underlying patterns and variations in crack propagation, forming a robust foundation for the models to develop predictive capabilities. Allocating 30% of the dataset to the validation set provided a significant portion of data for evaluating the trained models’ performance. A substantial test dataset was vital as it assessed the models’ generalization ability, indicating their performance on previously unseen data. This split strategy ensured a meaningful evaluation of the models’ predictive accuracy and instilled statistical confidence in their performance metrics. The 70/30 split struck a balance, addressing overfitting and underfitting issues.

**Figure 3:**
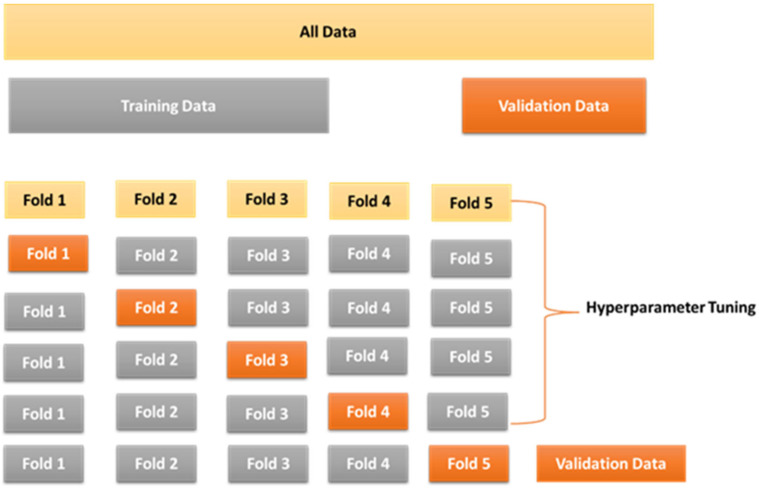
K-Fold cross-validation schematic.

To optimize model performance, hyperparameters were fine-tuned to minimize prediction error, ensuring satisfactory performance. Upholding the principle of testing on unseen data during training, a K-fold cross-validation methodology was employed, as shown in Figure 3. This approach guaranteed that the model underwent evaluation using entirely unseen data throughout the training process, preventing any compromise to its performance.

### 2.2. Feature Engineering and Model Selection and Configuration

The importance of feature engineering in understanding data cannot be overstated. When analyzing crack propagation in ABS materials, a specific set of features is meticulously chosen or engineered to accurately depict the material’s characteristics. Techniques such as correlation analysis and feature importance ranking are employed to identify the features that significantly influence crack propagation, as shown in the following sections. The H2O library offers a diverse range of machine learning algorithms tailored for regression tasks. Given the unique context of crack propagation, appropriate algorithms are selected from the offerings of H2O. Various model parameters, including learning rate, regularization strength, and the number of layers, are fine-tuned to optimize model efficiency while preventing overfitting. Validation methods, such as K-fold cross-validation, are utilized to assess the models and identify the most effective one. The H2O AutoML framework encompasses a wide variety of machine learning models, including XGBoost Gradient Boosting Machines (GBM), H2O GBM, Random Forests (default and Extremely Randomized Trees versions), Deep Neural Networks, and Generalized Linear Models (GLM). Notably, H2O is compatible with the popular XGBoost learning algorithm, allowing integration of third-party algorithms and enabling GPU-accelerated training. The current version of H2O AutoML can train and, if necessary, cross-validate a series of models, including three pre-defined XGBoost GBM models, an H2O GLM grid, a default H2O Random Forest, five specific H2O GBMs, a near-default H2O Deep Neural Net, an H2O Extremely Randomized Trees model, and random grids of XGBoost GBMs, H2O GBMs, and H2O Deep Neural Nets.

These pre-defined models serve as reliable benchmarks for each algorithm, providing room for user customization. The sequence of algorithms can be tailored, starting with consistently high-performing models such as pre-set XGBoost models, followed by a tuned GLM for immediate benchmarking. Subsequently, the algorithm introduces diversity by integrating a selection of Random Forests, GBMs, and Deep Learning models. After training and ranking these pre-set models, a random search commences across these algorithms. The time allocated for each algorithm during the AutoML run is predetermined, favoring certain algorithms such as XGBoost GBM and H2O GBM over H2O Deep Learning based on their perceived or calculated value. 

These techniques and methodologies are not limited to the current analysis but hold substantial potential for application in future research endeavors. They constitute a comprehensive toolkit for researchers, facilitating the exploration of diverse problem sets [36,37].

### 2.3. Model Training and Evaluation

The selected model is trained on the pre-processed dataset using H2Os training functions. The training process involves iteratively optimizing the model’s parameters using the training data. Model performance is evaluated using appropriate evaluation metrics, such as mean squared error (MSE), root mean squared error (RMSE), or R-squared. The dataset is split into training and testing sets to assess the model’s generalization ability and prevent overfitting. The model is iteratively refined by adjusting hyperparameters and retraining until satisfactory performance is achieved. To assess the accuracy and reliability of the automated model, several performance metrics are considered. In addition to MSE and RMSE, metrics such as mean absolute error (MAE), root mean squared logarithmic Error (RMSLE), and mean residual deviance are calculated. These metrics provide insights into the model’s ability to capture the variance in crack propagation behavior and make accurate predictions. Additionally, visualization techniques, such as scatter plots or residual analysis, are employed to further assess the model’s performance and identify potential areas for improvement. Through this methodology, we aim to develop an automated model using the H2O library that accurately predicts crack propagation in ABS materials. The dataset preprocessing, feature engineering, and model training processes ensure the model’s ability to capture the underlying patterns in crack growth behavior. The performance evaluation metrics provide a quantitative assessment of the model’s predictive capabilities, contributing to enhanced understanding and analysis of crack propagation in ABS materials.

## 3. Results and Discussion

### 3.1. Insights into Crack Propagation Behavior in ABS Materials

Analyzing the results from a comparative feature importance assessment involving multiple predictive models namely, Generalized Linear Model (GLM_1), Distributed Random Forest (DRF_1), and Gradient Boosting Machine (GBM_2), we uncover significant insights into the behavior of crack propagation in ABS materials. In this analysis, as shown in Table 2, each row of the table represents an individual feature, while each column corresponds to a distinct predictive model.

The constituent values within the table denote feature importance scores, typically calculated based on the contribution of each feature towards the model’s predictive output. Notably, a higher value suggests a more significant contribution of the corresponding feature to the prediction. Upon examining the comparative Table 1:

- The ‘amplitude’ feature demonstrates the highest importance for the GLM_1 and DRF_1 model, albeit less so for the GBM_2 model. This implies that amplitude variations significantly influence crack propagation in ABS materials, according to the GLM and Random Forest models.
- The ‘natural frequency’ feature, second in terms of importance for the GLM_1 model, emerges as the most crucial for the GBM_2 model. This feature also exhibits significant relevance for the DRF_1 model, suggesting that natural frequency plays a considerable role in understanding crack behavior across different models.
- The ‘temperature’ feature showcases lesser significance across all three models when compared with the ‘amplitude’ and ‘natural frequency’ features, indicating a relatively lesser role of temperature variations in the prediction of crack propagation behavior.
- The ‘crack location’ feature has been determined to be the least important amongst all the features across all the models, suggesting a limited role of crack location in influencing the crack propagation behavior in ABS materials according to these models. A noteworthy consideration is that feature importance does not indicate the direction (positive or negative) of a feature’s influence on the response but merely denotes the magnitude of its influence. Moreover, the scales of feature importance between different models may not be directly comparable due to the distinct methodologies each model employs for calculating feature importance. Thus, while comparing feature importance across models, the focus should be on the ranking of features within each model rather than on a direct comparison of the absolute values across models.

### 3.2. AutoML Models Selection

**Figure 4:**
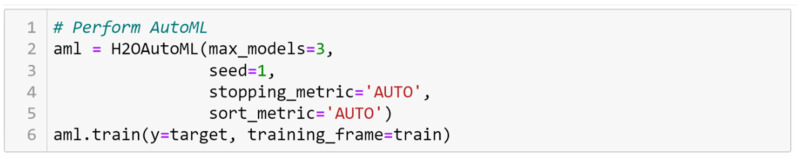
The parameters of AutoML operation.

The practical aspects of utilizing H2Os AutoML in Python and the significance of the corresponding code snippet cannot be understated. This segment of code initiates the AutoML process, furnishing vital parameters that steer the operation. In essence, it signifies the point of entry into H2Os robust automated machine learning capabilities. Critical parameters guiding the operation of the AutoML process include (see Figure 4):

- max_models: This parameter sets a limit on the number of individual models to train. By setting this to three, we ensure that the AutoML process will stop after training three models. This setting helps to control the computational resources and time spent during the AutoML process, depending on the complexity and size of the dataset.
- Seed: This parameter sets the seed for the pseudo-random number generator used by H2Os AutoML. It ensures that the randomness in the AutoML process, such as random hyperparameter selection, can be reproduced across multiple runs, improving the consistency and interpretability of our results.
- Max_runtime_secs: This parameter sets a limit on the total time spent in the AutoML process, in seconds.
- Stopping metric = ‘AUTO’ and Sort metric = ‘AUTO’: These parameters control the metric used to compare and rank different models. By setting these to ‘AUTO’, we allow H2Os AutoML to automatically choose the most appropriate metric based on the task.

Following the initialization of the H2OAutoML object, the AutoML process proceeds with hyperparameter optimization.

### 3.3. Hyperparameter Tuning and Training of AutoML Model

**Figure 5:**
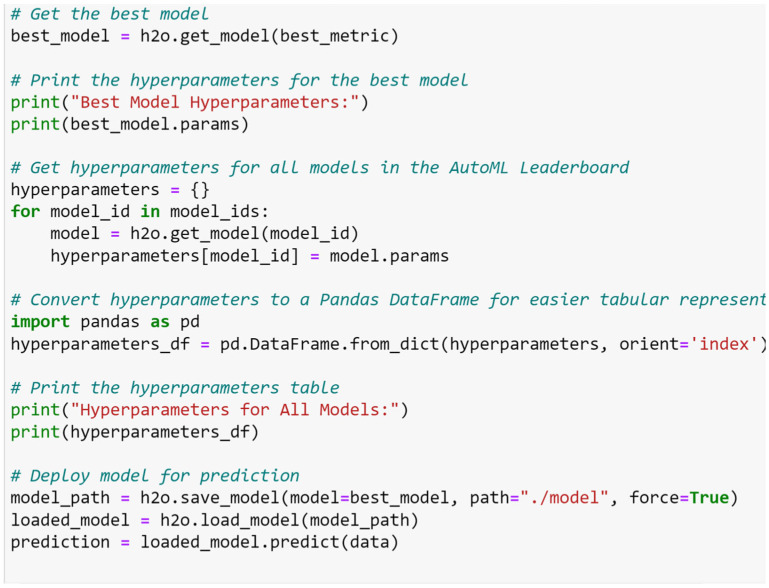
Code to identify optimal hyperparameters for all selected models.

Hyperparameter tuning, an essential process in ML, involves determining the optimal configuration for model performance. While traditional manual tuning can be resource- and time-intensive, recent advances in AutoML have significantly streamlined the process. This paper applied H2Os AutoML to a selected dataset, each with distinct features and complexities, to ensure the robustness of our results. A key aspect of our experimentation was the algorithm’s capacity for automatic hyperparameter tuning, which optimizes a model’s performance by fine-tuning its configuration. The underlying principle of hyperparameter tuning in H2Os AutoML is a method known as grid search. The framework builds multiple models with varying hyperparameters, facilitating the evaluation of different combinations to identify the one that yields the best model performance. Notably, the hyperparameter tuning process in H2Os AutoML is random, ensuring a diverse search space, and it automatically ranks models based on the selected evaluation metric. Following the hyperparameter optimization, H2Os AutoML proceeded with model training using the identified optimal hyperparameters, as shown in Figure 5. This ensured that the resultant models were not only theoretically optimized based on hyperparameter configurations but were also empirically validated through rigorous training processes. H2Os AutoML successfully executed both hyperparameter tuning and model training, resulting in the selection of optimal model parameters that yielded superior results, as shown in Table 3. 

This automated process significantly reduced manual intervention, minimized bias and human error, and resulted in a consistent improvement in predictive performance across various tasks. Moreover, the entire process, from data preprocessing to model training, was conducted within a reasonable timeframe, emphasizing the efficiency of the framework.

H2Os AutoML managed to simplify the process, making it accessible to both novice and experienced data scientists. This result aligns with the premise that the democratization of ML processes is a practical and necessary evolution, especially as the world becomes increasingly data-driven. The results demonstrate that H2Os AutoML process, with its integrated hyperparameter tuning and robust training mechanism, serves as a powerful tool in the ML toolkit, yielding high-performing models and simplifying the ML deployment process. Future work should explore the scalability of this approach for larger, more complex datasets and real-world applications.

### 3.4. Performance Evaluation of the Selected Automated Model

The developed automated model using the H2O library demonstrates promising performance in predicting crack propagation in ABS materials. Evaluations of the model’s accuracy were carried out using multiple performance metrics, as outlined in Table 3.

**Figure 6:**
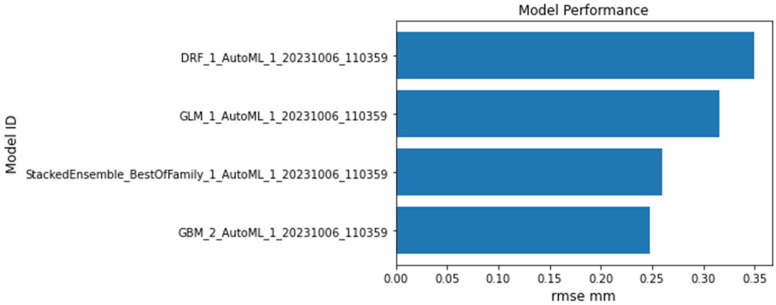
The performance of H2O-selected models is based on the RMSE metric.

The GBM model achieves an RMSE of 0.24784, an MSE of 0.0614249, a MAE of 0.209033, an RMSLE of 0.117522, and a mean residual deviance of 0.0614249. These values suggest that the GBM model offers the best performance among the models listed in terms of accuracy and fit, as shown in Figure 6. Conversely, the Stacked Ensemble model records an RMSE of 0.259332, an MSE of 0.0672531, and an MAE of 0.217328. The RMSLE value of 0.129487 and mean residual deviance of 0.0672531 further support its commendable performance, although it slightly lags behind the GBM model.

**Figure 7:**
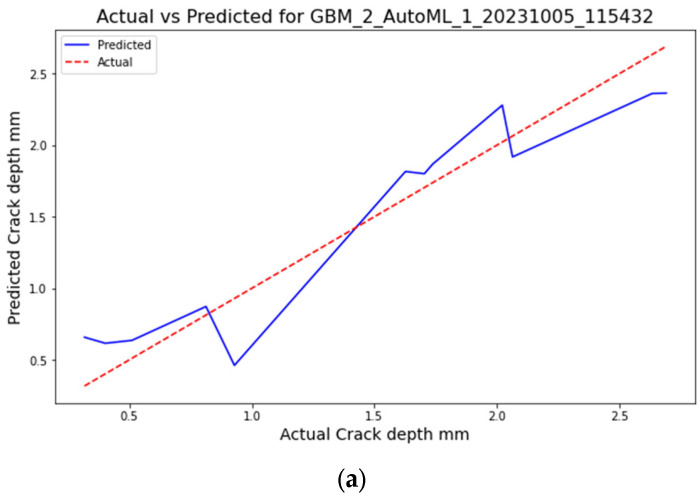

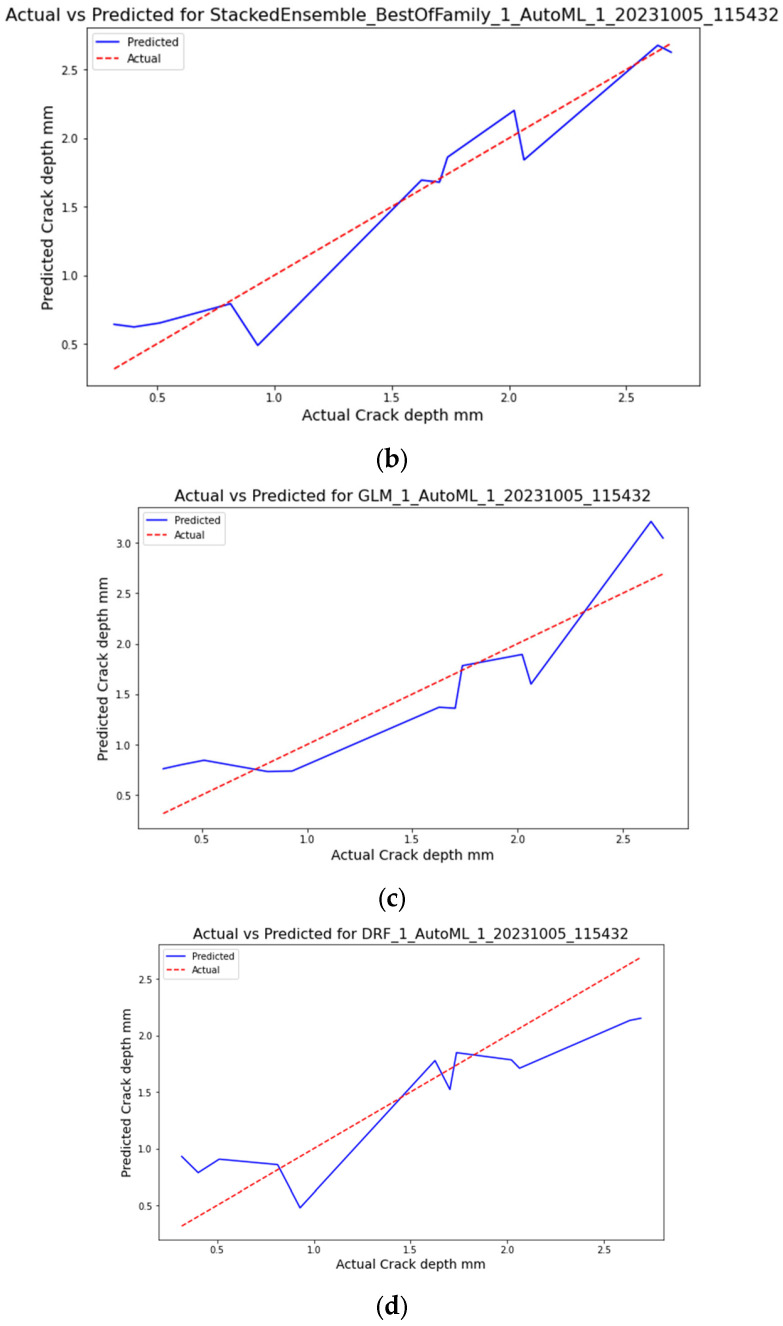
The actual versus predicted ABS crack depth (**a**). GBM AutoML (**b**). Stacked Ensemble Best of Family AutoML (**c**). CLM AutoML (**d**). DRF AutoML.

The DRF model exhibits higher RMSE, MSE, MAE, RMSLE, and mean residual deviance values of 0.349196, 0.121938, 0.297976, 0.162201, and 0.121938, respectively. These metrics suggest that while the DRF model is robust, it may not be as precise as the GBM or Stacked Ensemble models. The GLM model highlights RMSE, MSE, MAE, RMSLE, and mean residual deviance values of 0.315497, 0.0995384, 0.263245, 0.151175, and 0.0995384, respectively. Figure 7 illustrates the performance of the GBM, Stacked Ensemble, DRF, and GLM models over a series of iterations. It can be observed that the GLM model, although valuable, might have higher prediction errors compared to the other models, making it potentially less suitable for this dataset. The GBM model stands out as the most accurate based on the provided metrics, with all models offering valuable insights into crack propagation in ABS materials. These findings underscore the potential of machine learning approaches in structural health monitoring, setting a promising direction for future research.

### 3.5. Comparison with Machine Learning Approaches

H2Os AutoML is renowned for its advanced capabilities in tackling intricate predictive challenges. This tool harnesses a diverse range of machine learning algorithms, such as Deep Neural Networks, H2O GBM, GLM, and Random Forests. Additionally, it seamlessly integrates with the popular XGBoost model, facilitating the incorporation of external algorithms and GPU-powered training. The latest version of H2O AutoML is designed to train and, where required, cross-validate models. This includes a set of XGBoost GBM models, an H2O GLM grid, the standard H2O Random Forest, multiple specific H2O GBMs, a H2O Deep Neural Net that closely follows default settings, an H2O Extremely Randomized Trees model, and random configurations of XGBoost GBMs, H2O GBMs, and H2O Deep Neural Nets [38].

In contrast, conventional regression algorithms such as Linear Regression (LR), Back-Propagation Neural Network (BPNN), Classification and Regression Tree (CART), and Support Vector Regression (SVR) have been employed to forecast a range of material properties. This includes predicting the self-repairing ability of Engineered Cementitious Composite (ECC) [39]. These algorithms have demonstrated potential, with ensemble techniques such as bagging, AdaBoost, and stacking further enhancing prediction precision. Yet, they demand manual hyperparameter adjustments and might not be as adept as AutoML solutions in processing extensive datasets or intricate data interrelations. In research focused on forecasting crack growth in aviation aluminum alloys, the SVR algorithm was utilized. This model, trained using data from fatigue tests on crack length expansion, proved adept at accurately forecasting crack expansion between three holes [40]. In a separate investigation, reinforcement learning was deployed to determine the optimal times for structure inspections and decommissions to prevent failures. This research compared two distinct regression algorithms: neural networks (NN) and k-nearest neighbors (KNN) [41]. The results favored the KNN algorithm in terms of performance. While traditional regression techniques have yielded encouraging outcomes in predicting crack growth in diverse materials, AutoML solutions, such as H2O, present numerous benefits. These encompass efficient processing of vast datasets, automated hyperparameter adjustments, and the capability to discern intricate data patterns. Nonetheless, the selection of an algorithm should align with the task’s specific demands, such as dataset size and intricacy, interpretability necessities, and available computational resources.

## 4. Advantages of the Automated Model

The automated model developed using the H2O library for crack propagation prediction in ABS materials offers several advantages over traditional approaches. Firstly, the model demonstrates superior accuracy in predicting crack lengths, as indicated by the low RMSE and MAE values. This accuracy can significantly contribute to improving structural integrity assessment and maintenance strategies by enabling proactive measures to prevent catastrophic failures [42]. Secondly, the automated model reduces the dependency on manual intervention and subjective decision-making, as commonly encountered in analytical and numerical methods. By leveraging machine learning algorithms, the model can autonomously learn from the data and capture complex relationships, leading to more reliable predictions without the need for simplifying assumptions [38].

Additionally, the model’s efficiency and scalability are noteworthy. The H2O library provides efficient handling of large datasets and offers automated hyperparameter tuning, accelerating the model development process. The ability to process extensive datasets efficiently enhances the model’s robustness and widens its applicability to real-world scenarios [38,42].

## 5. Practical Implications

The application of the automated model to predicting crack propagation in ABS materials has significant practical implications for industries such as automotive, aerospace, and consumer goods manufacturing. Enhanced crack propagation analysis can lead to improved product design, material selection, and manufacturing processes, ultimately ensuring the safety and reliability of engineering components. The model’s predictive capabilities enable the identification of critical scenarios and the estimation of remaining useful life, facilitating more informed decision-making regarding maintenance and repair strategies. By accurately predicting crack growth behavior, proactive measures can be taken to mitigate potential risks, reduce downtime, and optimize maintenance costs. Furthermore, the insights gained from the automated model offer valuable guidance for materials scientists and engineers in the development of new materials and the optimization of existing ones. Understanding the influential factors on crack propagation in ABS materials can aid in the design of materials with enhanced fracture toughness, durability, and resistance to crack propagation, leading to improved product performance and longevity.

## 6. Limitations and Future Directions

While the automated model presents significant advancements in crack propagation prediction, it is important to acknowledge its limitations. The model’s performance heavily relies on the quality and representativeness of the training dataset. Inadequate or biased data may result in suboptimal predictions. Therefore, the availability of comprehensive and diverse datasets for model training and validation is crucial. Moreover, the model’s generalizability to different ABS materials and environmental conditions should be further investigated. The variability in material composition, manufacturing processes, and loading conditions may influence crack propagation behavior. Future research should focus on expanding the dataset to incorporate a broader range of ABS materials and considering additional factors such as temperature, humidity, and chemical exposure. Furthermore, the interpretability of the automated model warrants attention. Although machine learning models excel at predictive accuracy, understanding the underlying mechanisms and factors contributing to crack propagation can be challenging. Efforts should be made to develop explainable AI techniques that provide insights into the model’s decision-making process, allowing engineers and scientists to gain a deeper understanding of the crack propagation phenomenon. The automation of crack propagation prediction can be extended to other materials and structural components beyond ABS. The methodology presented in this study can serve as a foundation for developing automated models for different materials, allowing for broader applications in diverse engineering domains.

## 7. Conclusions

The automated model utilizing the H2O library presents a significant advancement in crack propagation prediction for ABS materials. The model’s accuracy, efficiency, and scalability offer substantial benefits for structural integrity assessment, maintenance strategies, and material design in various industries. The accurate predictions provided by the automated model enable engineers to make proactive decisions regarding maintenance and repair strategies, ultimately enhancing the safety and reliability of engineering components. By accurately forecasting crack propagation behavior, potential risks can be mitigated, downtime can be minimized, and maintenance costs can be optimized.

The efficiency and scalability of the automated model streamline the crack propagation analysis process. With the H2O library’s powerful capabilities, large datasets can be processed efficiently, enabling robust and reliable predictions. This efficiency allows for the analysis of extensive data sets, facilitating a comprehensive understanding of crack propagation behavior in ABS materials. Furthermore, the model’s ability to capture complex relationships between crack behavior and various influencing factors provides valuable insights into the underlying mechanisms of crack propagation. This deeper understanding enables engineers and scientists to make informed decisions regarding material design, manufacturing processes, and structural improvements, leading to enhanced product performance and longevity. While there are limitations and areas for future research, such as the need for diverse and representative datasets and the interpretability of the model, the automated model demonstrates the potential to revolutionize crack propagation analysis in ABS materials and beyond. Continued research and development in this field will further advance the automation of crack propagation prediction, enabling its application in a broader range of materials and contributing to enhanced safety, reliability, and performance of engineering components across industries.

The automated model utilizing the H2O library offers a powerful tool for accurate crack propagation prediction in ABS materials. Its impact extends to structural integrity assessment, maintenance strategies, and materials design, facilitating informed decision-making and advancing materials science and engineering practices. With ongoing research and development, the automation of crack propagation prediction will continue to drive improvements in safety, reliability, and performance in various industrial applications.

## Figures and Tables

**Table 1:** The experimental conditions and parameters.

Material	Temperature °C	Crack Location mm	Structural Response
ABS	50	5	Amplitude mm Natural Frequency Hz
	60	15	
	70	25	

**Table 2:** The importance of features for AutoML models.

Features	GLM_1	DRF_1	GBM_2
Amplitude	0.560706	0.439450	0.310581
Natural frequency	0.383545	0.534284	0.681861
Temperature	0.051501	0.012806	0.002864
Crack location	0.004248	0.013460	0.004693

**Table 3:** The performance of H2O models is based on different regression metrics.

Model	RMSE mm	MSE mm	MAE mm	RMSLE mm	Mean Residual-Deviance mm
GBM_2_AutoML	0.24784	0.0614249	0.209033	0.117522	0.0614249
Stacked Ensemble Best Off family	0.259332	0.0672531	0.217328	0.129487	0.0672531
GLM_1_AutoML	0.315497	0.0995384	0.263245	0.151175	0.0995384
DRF	0.349196	0.121938	0.297976	0.162201	0.121938
